# Vaping: An Emerging Health Hazard

**DOI:** 10.7759/cureus.7421

**Published:** 2020-03-26

**Authors:** Michael Oriakhi

**Affiliations:** 1 Internal Medicine, Medical Center Navicent Health, Macon, USA

**Keywords:** vaping, e-cigarettes, pulmonary disease, public health

## Abstract

Electronic cigarettes (e-cigarettes) are electronic devices designed to vaporize chemical compounds. The device is made up of a mouthpiece, liquid tank, a heating element, and a battery. E-cigarette use may pose health risks in the form of cardiovascular and respiratory diseases. These health risks have implications to not only the primary user, but the aerosols can also cause secondhand and thirdhand injuries to others in the vicinity. Acute lung injury may also be associated with the use of e-cigarettes, but the underlying cause remains unknown. Clinicians, including hospitalists, pulmonologists, intensivists, medical examiners, pathologists, and the like, should report possible cases as the medical community continues to assess the health risks of e-cigarette use.

## Introduction and background

The introduction of the new tobacco products and increased awareness has led to a dramatic rise in their use, especially by adolescents and young adults. This raises significant health concerns [1]. Simply put, electronic cigarettes (e-cigarettes) are electronic devices designed to vaporize chemical compounds. These devices have different components, including a mouthpiece, a liquid tank, a heating element, and a battery [2]. They come in various shapes, sizes, and device types, and are known by different names to different users. The most common terminologies include e-cigs, vapes, e-hookahs, vape pens, mods, tanks, or electronic nicotine delivery systems (ENDS). The process of using the devices is sometimes referred to as vaping or juuling, the latter so named for the particular device brand [3].

Even though they are called by different names, they all work on the same operating principle. It involves several steps. First, an electric current is activated either manually or automatically by a battery. Next, a current is delivered to the heating element. Finally, the liquid contained in the tank is evaporated and immediately condensed into a fine mist of liquid droplets (aerosols) [3].

The important difference is the substance contained in the device. It may contain varying substances. Some of these substances may be unknown and potentially harmful, especially when obtained from unauthorized sources (i.e., street sources) where they have been modified. Examples of these substances include nicotine, heavy metals (e.g., lead), volatile organic compounds, and carcinogenic chemicals [2,3]. These substances have the potential to cause severe respiratory and cardiovascular diseases.

## Review

So far, most of the literature on vaping-associated pulmonary disease we have are case reports and case series. A high index of suspicion is paramount as there are reports of patients that rapidly progressed to acute respiratory failure requiring intubation and mechanical ventilation [3]. The use of ENDS continues to grow in the United States. For example, while the use of combustible tobacco cigarettes has declined significantly, the United States ENDS market now exceeds 8 billion dollars [4]. At the moment, there are regular advertisements for e-cigarettes, promoting them as viable and safe alternatives to cigarettes smoking despite an absence of any studies to prove superior efficacy to conventional smoking cessation strategies already studied, such as nicotine replacement, bupropion hydrochloride, varenicline, and counseling [5]. E-cigarettes use may pose health risks in the form of cardiovascular and respiratory diseases. Tobacco cigarette smoking is the primary cause of preventable cardiovascular death in the United States, and smoking cessation has long been the focus of significant public health efforts. The rates of tobacco smoking in the United States have continued to decline and reached historic lows according to a Surgeon General report in 2014. However, with this decline, the use of electronic cigarettes, introduced in 2007, has markedly increased, especially among young people [6]. Healthcare providers should be on the alert for symptoms suggestive of acute lung injury secondary to vaping and remind patients that even though some e-cigarettes contain nicotine, they are currently not approved by the Food and Drug Administration as a cessation aid for smokers. Clinicians, including hospitalists, pulmonologists, medical examiners, primary care physicians, pathologists, and the like, are reminded to report possible cases [3,7]. People should consider not using e-cigarettes. This is especially the case for high-risk groups, including those without prior experiences, teenagers, pregnant women/nursing mothers, or adults who do not currently use oral tobacco products. These health risks have implications to not only the primary user, but the aerosols can also cause secondhand and thirdhand injuries to others in the vicinity. However, the regulations for public e-cigarette use vary across states and are inconsistent across cities within certain states. These variations in restrictions exist in both locations and types of product use, public versus private use, and types of products allowed in certain places [7]. Consumers in most states must be 18 years or older to purchase the device, although underage sales have been reported in retailers and online. The Food and Drug Administration has expressed concerns that certain flavored e-cigarettes are appealing to youth who may be unaware of the products’ addictiveness and some others who may have never tried a nicotine product [8]. There are many compounds in the aerosols and liquids and the selling point mostly used is that it can serve as a “Healthier” alternative to tobacco smoking even though the Food and Drug Administration has not approved this. The American Cancer Society discourages the dual use of electronic cigarettes and cigarettes because such use has not resulted in reduced exposures to the harmful effects of smoking [9,10]. Flavoring was considered by most users as the most important reason for vaping [10]. Over the past year, the Center for Disease Control has drawn attention to severe pulmonary disease associated with the use of electronic cigarette products. There have been reports of more than 200 cases associated with the use of these devices, using both known and unknown products [11]. The exact cause of these findings is still uncertain. Available data have been either case reports or case series. Some of the reported cases of e-cigarette-associated pulmonary illnesses include spontaneous pneumothorax, acute eosinophilic pneumonia, respiratory bronchiolitis-associated interstitial lung disease, hypersensitivity pneumonitis, organizing pneumonia, and acute exogenous lipoid pneumonia [12-14].

Layden et al., in a study done in Illinois and Wisconsin, gave a preliminary report that highlighted the most common presentations of patients with pulmonary illnesses related to e-cigarette use. Most of the patients in their study presented with shortness of breath, cough, and chest pain [12]. The severity of the illness varied from mild shortness of breath requiring oxygen supplementation via nasal cannula to severe debilitating respiratory failure requiring intubation and mechanical ventilation [12,13]. Five patients identified in July and August 2019 had acute lung injury associated with e-cigarette use. These patients were identified in two different hospitals in North Carolina, all were admitted for hypoxemic respiratory failure. All the patients reported a history of recent e-cigarette use. They were initially admitted for community-acquired pneumonia (CAP), but their symptoms worsened with conventional treatment for CAP. They were eventually diagnosed with acute exogenous lipoid pneumonia [13]. Another patient had presented with shortness of breath; investigations yielded a diagnosis of spontaneous pneumothorax. An 18-year-old patient had no history of cigarette smoking but endorsed daily use of e-cigarettes. The patient then had a recurrent spontaneous pneumothorax. He had no significant medical or surgical history to increase his propensity for spontaneous pneumothorax [14].

Laboratory investigations of most of the patients showed leukocytosis with a left shift, elevated erythrocyte sedimentation rate, and mildly elevated transaminases. Blood and sputum cultures, influenza screening, Legionella, and mycoplasma tests were negative [12]. Bronchoalveolar lavage was performed on some patients. These samples were tested using polymerase chain reaction and cultures. The results showed a mixture of neutrophils, lymphocytes, and lipid-laden macrophages. There were no bacteria, fungi, or viruses in the samples collected [13,15].

Chest computed tomography scans obtained from patients with vaping-associated lung disease showed significant findings. Ground-glass opacities seem to be a common finding in the imaging studies of most patients with e-cigarette-associated lung disease [16,17].

Some patients reported improvement in their symptoms with the cessation of vaping use [7,12,13]. While there were reports of rapid improvement with the use of systemic steroids, there was a report of a patient who, despite a high dose of systemic steroids and supportive therapies including extracorporeal membrane oxygenation therapy, died of acute lung injury [12,13,16.17].

Understanding the nature and attitude of e-cigarettes use among young adults is very important as this is the period when they transition into social contexts (e.g., college, peer pressure, and workplace) often resulting in an increased prevalence in substance use and the development of addictive patterns [18]. This raises a public health concern as it suggests that the younger generations of users, which have the highest rates of electronic cigarettes use, might become addicted to these devices despite unknown long-term physiologic and pathologic consequences [19].

## Conclusions

There is an increasing usage of electronic devices as more people are turning to it as an alternative to tobacco smoking. Other non-smokers are engaging in the use due to its increasing availability in social settings since its introduction to the United States. The increased use has also led to the increasing reports of vaping-associated pulmonary diseases. Various acute pulmonary illnesses have been reported as well as cardiovascular diseases. The presentation and complications vary amongst patients depending on the presence of other comorbidity. While there were reports of improvement with steroid use in some patients, most of the available regimens are still based on case reports and case series. Vigorous research is still ongoing to elaborate on the association between electronic cigarette use and acute lung injury. The details of the harm and extent of societal damage from the associated products are still ongoing.
